# Complete Genome Sequencing of the Novel Pseudomonas aeruginosa Phage UF_RH1

**DOI:** 10.1128/mra.00139-23

**Published:** 2023-05-11

**Authors:** Abdolrazagh Hashemi Shahraki, Majid Vahed, Mehdi Mirsaeidi

**Affiliations:** a Division of Pulmonary, Critical Care and Sleep, College of Medicine-Jacksonville, University of Florida, Jacksonville, Florida, USA; Loyola University Chicago

## Abstract

Here, we present the genome sequence of a novel Pseudomonas aeruginosa bacteriophage called UF_RH1. This lytic phage has a genome size of 42,567 bp and is classified as a member of the *Siphoviridae* family and the *Septimatrevirus* genus. UF_RH1 shares genetic similarities with *Stenotrophomonas* phage vB_SmaS-DLP_2.

## ANNOUNCEMENT

Pseudomonas aeruginosa is an opportunistic pathogen that poses significant challenges for treatment (1). However, one effective approach to combat this pathogen is phage therapy, particularly with lytic phages (2). A P. aeruginosa phage named UF_RH1 was isolated by introducing 10 μL of filtered wastewater collected from a sewage treatment plant (Alexander Orr Water Treatment Plant located at Miami, FL) to 400 μL of P. aeruginosa (strain DJ06) in logarithmic phase and then performing single-plaque isolation using double-layer agar (3). The purity of the phage was confirmed after five rounds of single-plaque isolation. Phage DNA was extracted using a commercial kit (QIAamp DNA Mini kit; Qiagen, USA). The Nextera XT library preparation kit (Illumina, San Diego, CA) was used to create a phage DNA library, which was sequenced on the Illumina NovaSeq 6000 platform with 150-bp paired-end reads. The raw reads were cleaned up with the Cutadapt program v2.8 (4) to remove low-quality bases and sequencing adaptors and then searched against the genome of P. aeruginosa from the NCBI genome database using the read mapper of the STAR package to remove potential host DNA contamination (5). The unmapped paired-end reads were further assembled with the software MetaWRAP v1.2.0 (6), and the assembled consensus sequences with a length of more than 5,000 bp were evaluated by QUAST v5.0.2 (7) and analyzed with the metagenomic software Centrifuge v1.04b (8). The quality of the viral genome completeness and identification of closed genomes were assessed using the CheckV v1.01 package (9). The viral genome candidates were further analyzed with NCBI BLASTn to determine the taxonomic relationships of the phage (10), and Victor was used for phylogenetic analysis (11). Open reading frames (ORFs) were identified by GeneMarkS (12), and the genome was annotated based on PHASTER (13) and BLASTp results (10). PhageTerm was used to determine the phage termini (14). The tRNA sequences were determined by tRNAscan-SE v2.00 (15), while virulence factors and antibiotic resistance factors were detected using ResFinder v4.00 (16) and the Antibiotic Resistance Genes Database (17), respectively, with default parameters for all software tools.

**FIG 1:**
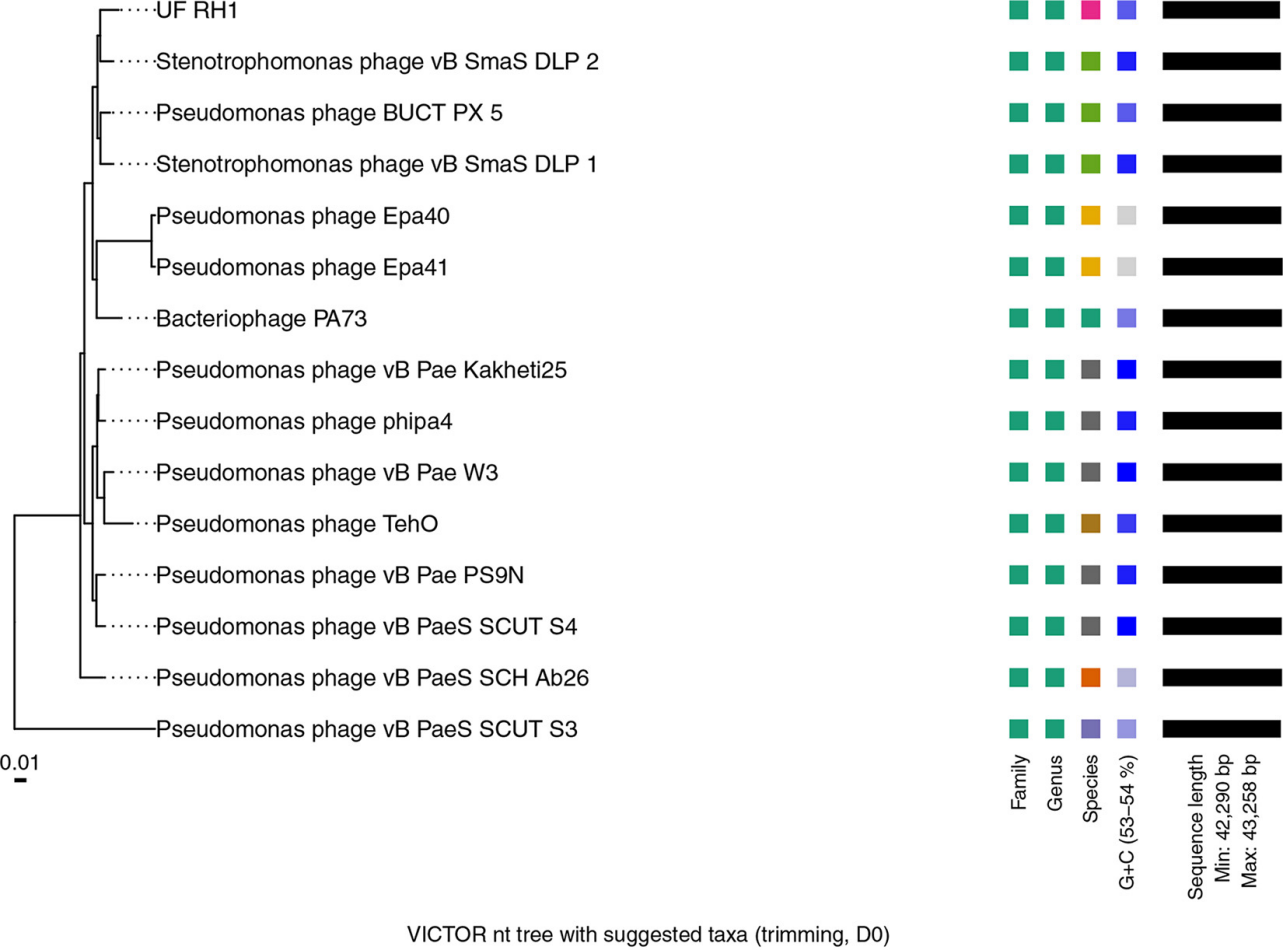
Whole-genome-based phylogenic tree (with average nucleotide identity grouping) and taxonomic position of UF_RH1 among closely related phages of genus *Septimatrevirus*. Three series of color boxes behind the tree indicate viral taxonomy position (family, genus, and species), and the last one shows the GC content (%) for each virus.

The genome of Pseudomonas phage UF_RH1 is a liner double-stranded DNA (dsDNA) genome with a length of 42,567 bp, a GC content of 53.56%, 931,200 total reads, and 23,360× average read coverage. PhageTerm predicted a circularly permuted genome for UF_RH1. It includes 57 open reading frames (ORFs), and it shares sequence similarity with members of the genus *Septimatrevirus* (Fig. 1). *Stenotrophomonas* phage vB_SmaS-DLP_2 (18) is a closer phage to UF_RH1 (Table 1). The annotated proteins of UF_RH1, such as dead box helicase, RecB exonuclease, central tail hub, and holin, exhibit 99%, 98%, 98%, and 86.36% similarity, respectively, to the corresponding annotated proteins of *Stenotrophomonas* phage vB_SmaS-DLP_2. DNA polymerase and endolysin also show the highest similarity of 99.8% and 92%, respectively, to the proteins encoded by Pseudomonas phage TehO. The tail length tape-measure protein also displays the highest similarity of 99.58% to the tail protein of Pseudomonas phage vB_PaeS_C1. UF_RH1 does not contain tRNA, virulence, and antibiotic-resistant genes.

**TABLE 1:** Genome sequence coverage and nucleotide identity of UF_RH1 with their closest relatives^*a*^

Phage (GenBank accession no.)	Sequence coverage (nucleotide identity) of:							
	UF_RH1	vB Pae-Kakheti25	vB SmaS-DLP_2	Epa41	Epa40	phipa4	vB PaeS_SCUT-S4	PA73
UF_RH1 (OQ259603.1)	100 (100)	95 (96.2)	97 (97.8)	92 (94.8)	91 (94.8)	95 (94.1)	94 (97.2)	94 (94)
vB Pae-Kakheti25 (JQ307387.1)	95 (96.2)	100 (100)	95 (97.08)	91 (96.8)	90 (96.8)	97 (97.9)	98 (96.8)	93 (97.6)
vB SmaS-DLP_2 (KR537871.1)	97 (97.8)	95 (97.08)	100 (100)	89 (97.36)	89 (98.37)	95 (97)	95 (97.3)	93 (98.33)
Epa41 (MT118305.1)	92 (94.8)	91 (96.8)	89 (97.36)	100 (100)	99 (100)	89 (97.5)	89 (98.34)	92 (97.67)
Epa40 (MT118304.1)	91 (94.8)	90 (96.8)	89 (98.37)	99 (100)	100 (100)	89 (97.5)	89 (98.34)	92 (97.75)
phipa4 (OK539825.1)	95 (94.1)	97 (97.9)	95 (97)	89 (97.5)	89 (97.5)	100 (100)	98 (96.88)	94 (98)
vB PaeS_SCUT-S4 (MK165658.1)	94 (97.2)	98 (96.8)	95 (97.3)	89 (98.34)	89 (98.34)	98 (96.88)	100 (100)	91 (97.64)
PA73 (DQ163913.1)	94 (94)	93 (97.6)	93 (98.33)	92 (97.67)	92 (97.75)	94 (98)	91 (97.64)	100 (100)

a All values are %. All phages are classified as Pseudomonas phage except vB_SmaS-DLP_2, which is classified as *Stenotrophomonas* phage.

### Data availability.

The complete phage genome sequence was deposited in GenBank under the accession number OQ259603. The raw data are available in the NCBI Sequence Read Archive (SRA) under BioProject accession number PRJNA936202, SRA accession number SRS16838829, and BioSample accession number SAMN33343989.
